# Book Review

**Published:** 1901-09

**Authors:** 


**Diseases of the Thyroid Gland.** Part I.—*Myxœdema and Cretinism.* By George R. Murray, M.D. Pp. viii., 112. London: H. K. Lewis. 1900. No one can speak with greater authority on the subject-matter of this little monograph than the distinguished author. Some of the contents have already appeared in print, for nearly the whole of the Goulstonian Lectures on the pathology of the thyroid gland have been incorporated in the volume, as well as some extracts from Dr. Murray's article on "Diseases of the Thyroid Gland" in the *Twentieth Century Practice of Medicine.* Nevertheless, many practitioners and students will be glad to have these writings in a collected and expanded form. The work is a complete and convincing description of myxœdema and cretinism, and their treatment. We note that Dr. Murray favours the use of thyroid extract, either in the form of the liquid extract, the dry extract, or of the fresh gland itself, rather than thyroidin or other preparations which have been put forward as the active principles of the thyroid gland tissue. We strongly commend the book as a complete and moderate statement of what may be done by the timely exhibition of thyroid gland tissue in suitable conditions.

**The Norfolk and Norwich Hospital, 1770 to 1900.** By Sir Peter Eade, M.D. Pp. 253. London: Jarrold & Sons. 1900. —This very complete record of the history of a large provincial hospital, from its foundation in 1771, to its demolition and rebuilding in 1883, with a further account of the present hospital up to the present date, contains much information of great interest, not only locally and to its own staff, but to all who are concerned in the management of hospitals elsewhere. Amidst the controversies surrounding the question of the representation of the medical staff on the board of management, the author gives us the information that "at the present time, not only is the advice of the Honorary Medical Staff very frequently asked, and their presence at the Weekly Board welcomed, but the whole of their number are *ex officio* members of this Board, and four of them (elected annually by themselves) have votes upon it. Whilst at the same time the Honorary Consulting Officers are Life Members of the Board, with all privileges." The hospital has manifestly not suffered from so large and liberal a representation of the working staff, whose presence, when time can be spared from their more active medical and surgical duties, cannot fail to be of assistance to the lay governors. The present hospital occupies an area of eight acres, just outside the city: it is built on the separate pavilion principle, connected by corridors, and two-storied. The hospital is built for and contains 214 beds; each of the large wards has 24 beds, and there are eight of these. The flooring is of pitch-pine, and the whole hospital is lighted by electricity. The annual expenditure amounts to nearly ten thousand pounds. Patients are admitted by letter of recommendation, but we are glad to observe that the laws upon this point are liberally interpreted, inasmuch as the "number of in-patients admitted without recommendations was, last year, 600, as against 932 recommended by subscribers." We hold strongly to the view that urgent cases demanding active surgical treatment should not be required to exhaust themselves and their friends in the search for letters of recommendation, when the case itself is a sufficient and obvious recommendation, and is so recommended by any member of the surgical and medical staff. We congratulate the author on his excellent account of an institution on which every care has been expended to make it as complete and efficient as is possible, which has a notable history extending considerably over a century, and which will compare well with the hospitals of the new century for many years to come.

**Lessons in Elementary Physiology.** By THOMAS H. HUXLEY, F.R.S. Enlarged and Revised [Fifth] Edition, [by M. FOSTER and S. LEA.] Pp. xxiv., 611. London: Macmillan and Co., Limited. 1900.—For many years after the first appearance of Huxley's *Physiology* in 1866 it continued to rank as one of the standard text-books on the subject. Its popularity may be judged of by an inspection of the long list of reprints appearing since the first edition was published. The fourth edition appeared in 1885. Since that time physiology has made enormous strides, and it speaks eloquently for the merit of a book that it should still be found useful when thirty-five years old, even when dealing with a rapidly growing science. But the book itself is an altogether exceptional one. It was written by a man who was at once an enthusiast and a master; indeed, it is not too much to say that Huxley was the very founder of scientific biology in this country. And so the master's book has continued in use till to-day; and now that the whole of the science has changed so greatly, the task of preparing a new edition has been entrusted to the hands best qualified in England to carry out the work. The old familiar form of the "lessons" has been preserved, and the simple form of exposition which appealed so forcibly to young students still appears, but the whole has been brought up to date and many new illustrations have been introduced. We may perhaps best express our opinion of the book by saying that if we had our way every student of physiology would be made to read, mark, learn, and inwardly digest Huxley's "lessons" before proceeding to the study of a more ambitious work.

**Cyclic Albuminuria.** By G. A. SUTHERLAND, M.D. Pp. vii., 63. London: The Medical Publishing Company, Ltd. 1900.—This is a useful monograph on a subject of interest for life assurance as well as on scientific grounds. True cyclic albuminuria (Pavy'sche Krankheit) is not the only form of functional albuminuria known, but it is the most common type in early adult life. The discussion is based on fifteen cases minutely watched by the author, who reviews much of the literature which has grown up on the subject since the disease was defined by Pavy in 1885. He does not believe that it is the result of a previous nephritis, but rather due to a condition akin to lithæmia, and comparable to the intermittent albuminuria seen in Graves' disease, where also some toxin probably exists in the blood. Casts and other evidence of nephritis are absent, and albumen itself is absent so long as the patient remains in bed, but appears soon after rising and declines during the afternoon. On the whole he finds no evidence that the affection is an early stage of or leads to Bright's disease, though there are few facts known as to the final history of these patients. The sufferers are probably neurotic, and a quiet, healthy life is advisable for them, free from anxiety and strain. When accepting them for insurance it is at present necessary to be sure that the albumen has disappeared altogether, and that there is no evidence of cardiac or renal changes and no history of scarlatina.

**Heart Disease in Childhood and Youth.** By CHARLES W. CHAPMAN, M.D. Pp. 101. London: The Medical Publishing Company, Limited. 1900.—The subject of cardiac disease in children is an important one. This book is, however, more a collection of cases than a work upon this subject. Collections of cases always possess a certain value, and this collection possesses some cases of interest, but not many that seem to call for special comment. In one a systolic apical murmur is described which "came and went" without apparent cause. We can hardly believe that this was endocardial. Much of the advice given is sound common sense, but the book can scarcely be said to throw great light upon cardiac disease in children.

**Practical Uranalysis and Urinary Diagnosis.** By CHARLES W. PURDY, M.D. Fifth Edition. Pp. xvi., 392. Philadelphia: F. A. Davis Company. 1900.—This book has so quickly reached a fifth edition, and we reviewed it so recently, that it is not necessary to say more than that it retains its chief features, but has been thoroughly revised, and, in our opinion, much improved. The tests for albumin are given more fully, and in a more satisfactory manner: the four tests which the author considers most useful are given in large, the others in small, type; but it would be useful to the beginner if the author would indicate clearly the exact order in which the tests should be employed in order to use them in the most scientific way. The author has further developed his method of estimating albumin, chlorides, phosphates, sulphates, and other bodies in the urine by means of his electrically-driven centrifuge, and claims for it accurate quantitative results if his directions are followed out. The method is rapid and simple, and would seem to be a great improvement in these respects on the more tedious methods of quantitative analysis in general use. A description of the microscope which has been added seems to us now-a-days an unnecessary addition to a book on the urine, but the section on the method of microscopic examination of urinary sediments will be found useful to the student, and a paragraph might be added with advantage on the best method of making permanent preparations of sediments. On page 368 the pulse in chronic Bright's disease is said to be "full, hard, and unresisting to the finger:" surely incompressible is the word meant. We can only say again that the book is a good practical guide to the subject, sufficiently illustrated, and fully deserves the success it has gained.

**Records from General Practice.** By J. Kingston Barton. Part II. Pp. 85—208. London: John Bale, Sons, and Danielsson, Ltd. 1901.—We can recommend this book to anyone wishing to while away an odd hour with light medical reading. The author chats pleasantly of cases he has met with, and of remedies that he has found useful, etc.; but when it comes to accepting the deductions he draws from his experience —or rather the impressions that he has formed as he pursued his practice—it is quite a different matter, for some of his conclusions are founded on very insufficient data and are certainly not above criticism.

**American Medicine.** Vol. I. Nos. 1 and 2. 1901. Edited by George M. Gould. Published Weekly. Philadelphia: American Medicine Publishing Company.—The editor's name is alone a guarantee that this new weekly medical journal is worthy of American medicine, and we desire to give a cordial welcome to our new contemporary. The journal is founded, owned, and controlled by members of the medical profession in America, and the two numbers before us contain many very practical yet scientific articles, mostly by physicians or surgeons with whose names we are already familiar. It is freely illustrated, and the printing and editing leave little to be desired. Notwithstanding the fact that amidst numberless medical journals hailing from America there are already several which take the highest rank in the world's literature, we believe that *American Medicine* will take a foremost place and be widely read, for it has distinctive features and is well suited to the daily wants of busy practitioners.

**How to Avoid Tubercle.** By Tucker Wise, M.D. Third Edition. Pp. 24. London: Baillière, Tindall & Cox. 1900.—This, the third edition, has grown considerably larger since our former notice. We find that the cat and dog are still allowed to remain, but must be "properly fed and looked after, and in case of suspected illness sent off at once to the veterinary surgeon." The author still advises that the sputum shall be thrown down the w.c. Why should it not be burnt, and so once for all effectually destroyed? We notice that the habit of spluttering in conversation should be cured without loss of time.

**The Harveian Lectures on Prognosis and Treatment of Pulmonary Tuberculosis.** By ROBERT MAGUIRE, M.D. Pp. 48. London: Baillière, Tindall and Cox. 1901.—This reprint from *The Lancet* of December, 1900, contains the latest views on the foundations for prognosis, and reviews the various methods of treatment in vogue. The author concludes with a preliminary announcement on the intra-venous injection of formic aldehyde, from which he expects great results, if his experiments on himself do not put a stop to his investigations.

**An Index of Symptoms.** By RALPH WINNINGTON LEFTWICH, M.D. Second Edition. Pp. xvi., 267. London: Smith, Elder & Co. 1901.—One feels sorry, on looking through this work carefully, that it is not likely to be one of much use to the student. The enormous trouble which it must have taken to compile is at once evident. It is made up of long lists of diseases under every conceivable symptom. There are about 240 double-column pages of these. "General wasting" may be due to one of 55 diseases; "pallor of the face" to 48; "headache" to 110, and so on. There is no doubt, therefore, that the list is comprehensive. One might also safely say that almost nothing has been left out. If a diagnosis is to be made in a mechanical way, by someone who knows almost nothing of medicine, then to them the book may be recommended; but otherwise, it would probably be an unprofitable labour to try and elucidate a case by its means. It is so often not merely the symptom that is of importance, but the conditions under which that symptom arises—as for instance, the age and sex of the patient, the degree and variety of the symptoms, and so on; but there is nothing here to show any such distinction. The idea is good, and some day this work as a pioneer may bring forth fruit, but at present it has mostly run to leaves.

**Arsenical Poisoning in Beer Drinkers.** By T. N. KELYNACK, M.D., and WILLIAM KIRKBY, F.L.S. Pp. xii., 125. London: Baillière, Tindall and Cox. 1901.—This book gives a careful account of arsenical poisoning as met with in the recent outbreak in the North of England. It opens with a short history of the epidemic and the persons chiefly affected by it; then follows a full description of the symptoms produced. The skin lesions, from their extent, number and intensity, are worthy of special notice, and are illustrated by some good plates. Two other points to be remarked upon are the severity of the sensory disturbances in this form of neuritis and the frequency of herpetic eruptions. There is, however, probably no danger that a case of arsenical poisoning will be overlooked for some time to come. The authors seem to establish the fact that many people moderate in the use of alcohol were affected, and that the outbreak was by no means confined to topers. It seems in the light of this occurrence that the views held as to the pathology of alcoholic neuritis will require careful sifting. The book has a chemical section on the tests to be employed for the detection of arsenic in beer, and concludes with a useful bibliography. Those who desire a clear account of the effects of arsenic can be recommended to read this book; the illustrations are good, and help to elucidate the clinical descriptions.

**Surgery: its Theory and Practice.** By WILLIAM JOHNSON WALSHAM. Seventh Edition. Pp. ix., 953. London: J. & A. Churchill. 1900.—When a work has reached a seventh edition, it is, perhaps, less open to criticism than a first edition. "Walsham" has become a big book in a small compass, and its utility has suffered by the inclusion of much out-of-the-way matter to the exclusion of adequate detail on important subjects. Thus, amputations are described in an appendix of eight pages with only five illustrations, whilst four illustrations are given to the reduction of dislocations of the hip by pulleys—a method which we doubt is ever employed in the present day. Malgaigne's hooks for fractured patella are deemed worthy of illustration to impress the student's mind. We doubt whether bleeding from an artery in its continuity should be treated by a "thorough trial" of "pressure applied at the seat of wound" (p. 128) before resorting to ligation; or that the average man should resort to Davy's lever to compress the common iliac *per rectum;* or that ligation of the brachial artery, or of the radial in its upper third, can be adequately described in six lines (pp. 314, 315). "Looseness of the capsule" (p. 444) can hardly explain the frequency of dislocations of the shoulder. We were not aware that the "coronoid process is often fractured" (p. 452) in dislocations at the elbow-joint, and we think the advice, "Passive movements should be cautiously begun about ten days or a fortnight after the injury" (p. 454) to be unwise conservatism. In cases of acute intestinal obstruction it is stated "nothing whatever should be given by the mouth" (p. 703); a few lines later, it is said "a pint or so of hot water may be given" (p. 705). We regard as dangerous the recommendation to explore the sinus and remove the appendix when a fistula persists after operation for suppurative appendicitis; and to dilate strictures of the rectum by bougies without a word of caution being given (p. 767). The skiagraphic additions are excellent, with few exceptions. The illustrations are numerous, generally good, but a few are poor. Fig. 321 is particularly unhappy. Laryngoscopy there appears like the administration of a dose of jalap to an unappreciative recipient. On page 376 "aseptic pneumonia" should read "a septic pneumonia." On the whole there is much to commend, but a great lack of proportion in the book.

**Vasectomy and Urethro-Stenosis.** By REGINALD HARRISON. Pp. 68. London: J. & A. Churchill. 1900.—This book is simply a reprint of papers which have been contributed by the author to the *Lancet*, and of a report to the International

Medical Congress of 1900 on "Urethro-Stenosis." Throughout the work the personal element is strong, and it culminates in a catalogue of the author's published works and papers, occupying eight pages, which we think out of place in a book of this kind. We presume "The Use of the Ambulance in Civil Practice" has no conceivable connection with enlarged prostate or stricture.

**Diseases of the Tongue.** By Henry T. Butlin and Walter G. Spencer, M.B. Pp. 475. London: Cassell & Company Limited. 1900.—Fifteen years have elapsed since Mr. Butlin first presented us with his excellent manual on this subject. During this interval the knowledge of diseases of the tongue has greatly increased, and the author speaks with a more authoritative voice upon questions which have been answered by a ripened experience. This is perhaps most noticeable in the excellent chapters dealing with the early manipulation of cancer and the operative treatment necessary for the complete eradication of the disease. Mr. Butlin admits that there is yet much to learn of the relationship between cancer of the tongue and the lymphatic glands in the neighbourhood, but he rightly insists on the desirability of clearing out the glands in the anterior triangle subsequent to the removal of the local malignant growth. In the preparation of this second edition he has been ably assisted by Mr. Spencer, who is more particularly responsible for the chapter on the anatomy of the tongue and for much of the more recent pathology. The book contains some very good chromo-lithographs, and though arranged on the same general lines as the former edition, must be largely regarded as a new work. The authors are to be congratulated upon the production of a standard treatise of an exhaustive and original character.

**The Goulstonian Lectures on Typhoid Bacillus and Typhoid Fever.** By P. Horton-Smith, M.D. Pp. 109. London: J. & A. Churchill. 1900.—These lectures afford a full *résumé* of the present-day knowledge of the bacteriology of enteric fever. The first is devoted to a description of the life history of the organism and its distribution in the body. The second deals with the varieties and complications of the disease, and the third with a critical survey of Widal's reaction. The author lays especial emphasis upon the infective nature of the urine in enteric fever, and on the value of urotropin in securing its sterility. The appendix, detailing cases of typhoid bacilluria and cystitis, will well repay study.

**Angioma, and other Papers.** By the late John Duncan. Edited by James Hodsdon. Pp. xvi., 177. Edinburgh: Oliver and Boyd. 1900.—It is refreshing to read these interesting papers by one who is described as "a manly man—broad-minded and whole-hearted." The papers on angioma and on electrolysis in aneurysm are particularly worth reading; the former illustrating the author's powers of observation, and the latter the results of a well-balanced enquiry into the value of electricity in the treatment of aneurysm.

**The Extra Pharmacopœia.** By William Martindale and W. Wynn Westcott, M.B. Tenth Edition. Pp. xxxii., 688. London: H. K. Lewis. 1901.—It is only two years since we were called upon to notice the ninth edition of this indispensable volume, which grows steadily larger, and contains the names of far more drugs than are needful for the patients who wish to take them. Abstracts have been incorporated from the Indian and Colonial addendum to the B.P., 1898, as also from the formulary of the British Pharmaceutical Conference. One rarely fails to find any information which may be required, and the book must be a constant companion to the dispensing chemist as well as to the physician who prescribes.

